# Towards scalable age-grading of *Aedes albopictus* mosquito using mid-infrared spectroscopy and machine learning

**DOI:** 10.1038/s41598-025-24404-x

**Published:** 2025-11-18

**Authors:** Mattia Foti, Martina Micocci, Mauro Pazmiño-Betancourth, Ivan Casas Gomez-Uribarri, Paola Serini, Beniamino Caputo, Alessandra della Torre, Francesco Baldini

**Affiliations:** 1 Department of Public Health and Infectious Disease, University of Rome La Sapienza, Rome, Italy; 2 School of Biodiversity, One Health, and Veterinary Medicine, University of Glasgow, Glasgow, UK; 3 Department of Environmental Health and Ecological Sciences, Ifakara Health Institute, Ifakara, Tanzania

**Keywords:** Computational biology and bioinformatics, Mathematics and computing

## Abstract

The age structure and dynamics of mosquito populations are crucial for understanding their ability to spread diseases and assessing the effectiveness of anti-mosquito control measures. However, available methods to age-grade mosquito populations are labour-intensive and imprecise, particularly for *Aedes* species. We investigated the potential of Mid-Infrared Spectroscopy (MIRS) combined with Supervised Machine Learning (ML) to rapidly and accurately predict the age of adult females and males of the arbovirus vector, *Aedes albopictus*. First, we demonstrated the ability of MIRS-ML to age male and female mosquitoes reared under laboratory conditions. Second, we optimised the model with adults emerged from wild collected eggs reared under natural conditions in a semi-field facility, to expose them to more realistic ambient conditions. For each sex we developed three ML models based on the resolution of the predicted adult age class: low (grouping of the mosquitoes by age in 9-day interval), medium (6 days) and high resolution (3 days) from 1 to 15 or 33 days for males and females, respectively. The prediction accuracy decreased as the resolution increased. In males, the accuracy dropped from 99% (low resolution model) to 93% (medium resolution model) and 85.8% (high resolution model); in females the low and medium resolution models showed 89.4% and 78.5% accuracy, which decreased to 72.6% for the high resolution. In a simulated vector control intervention, the high-resolution models allowed to detect shifts in the age-structure of *Ae. Albopictus* populations with minimal sampling effort (< 100 specimens). Finally, we validated MIRS-ML on two unseen data and reconstructed plausible age structures in (1) laboratory-reared and (2) field-collected *Ae. albopictus* males and females. Overall, the results represent a first step towards the development of a sound and reproducible MIRS-ML approach for age-grading of *Ae. albopictus* populations in the wild.

**Supplementary Information:**

The online version contains supplementary material available at 10.1038/s41598-025-24404-x.

## Introduction

Accurately estimating the age structure of wild mosquito populations can significantly improve assessments of disease transmission risk and the effectiveness of vector control interventions^1–3^. However, determining mosquito age remains a challenging task, especially when relying on limited sampling efforts.

Traditional methods for estimating age of female mosquitoes rely on assessing ovary morphology changes associated with gonotrophic cycles^4,5^. These approaches are labour-intensive, technically demanding, difficult to standardise, and provide only indirect and often imprecise estimates of biological age^1,6^. Moreover, they are primarily developed for *Anopheles* species, and show limited reproducibility when applied to other genera, such as *Aedes*^7,8^, making them unsuitable for broader surveillance applications. In recent years, alternative age-grading techniques have emerged, including those based on proteomics, gene profiling and gene expression. These have been applied mainly to major *Anopheles* vectors of malaria in sub-Saharan Africa^9,10^ and the arbovirus vectors such as *Aedes aegypti* and *Aedes albopictus*^10–13^. However, despite their promise, these molecular approaches face significant barriers to large-scale implementation due to high costs and a lack of conclusive studies validating their reproducibility. As a result, none of these methods has yet been established as a gold standard for mosquito population age-grading^14,15^.

In recent years, infrared spectroscopy (IR) has been proposed as an alternative for mosquito age-grading based on the detection of age-related changes in the chemical composition of the mosquito’s cuticle^16^. By directing light onto a sample, IR quantifies the energy absorbed by molecules based on their vibrational modes, providing chemical spectra within seconds and without the need for any reagent^17,18^. IR requires minimal training personnel, and with the easiness of the spectral acquisition, a laboratory can process up to 50 samples per hour with one machine. The machine costs ~ $20,000, but does not require any reagents or running costs except for electricity. Thus, MIRS is cost-effective and less labor-intensive compared to molecular/immunological techniques^19,20^. The age- related spectral changes are then disentangled by machine learning algorithms, despite the subtle biochemical differences between specimens with different ages^21^. Both Near-Infrared Spectroscopy (NIRS, wavenumbers in the range 10,000 to 4000 cm^−1^) and Mid-Infrared Spectroscopy (MIRS, from 4,000 to 400 cm^−1^) have been proposed for age-grading Afrotropical malaria vector species of the *Anopheles gambiae* complex^22,23^, as well as for species identification^23^, pathogen detection in vectors^24^ and humans^25^ and blood meal identification^26^. Both NIRS and MIRS have been also successfully tested for age- grading *Ae. aegypti* and for detection of *Wolbachia* infection in laboratory samples of this species^27,28^.

Overall, MIRS shows a higher mechanistic robustness as it provides distinct and well-defined absorption bands corresponding to fundamental molecular vibrations, crucial to enable the generalisation of age-grading predictions across different settings^21,29^.

Less effort has been so far invested in the development of age-grading secondary vectors, such as *Ae. albopictus*, despite in the last decades, this highly invasive species has increased its public health relevance at the global level. In fact, in addition of being a secondary dengue virus vector and a primary one of Chikungunya virus in tropical regions, it has been responsible of the first autochthonous cases and outbreaks of these arboviruses in Europe^30–32^. We could not find published research that measured the accuracy of morphological age-grading methods in this species, and attempts made in Sapienza University to test these approaches on laboratory-reared females of known age revealed very low accuracy (data not shown). Preliminary attempts on *Ae. albopictus* age-grading by NIRS showed limited success, with high accuracies in age predictions obtained only for laboratory specimens < and > 7 days old^33,34^.

To our knowledge, MIRS has never been tested for age-grading *Ae. albopictus*. Here, we report the results of the first steps towards the development of a MIRS-ML framework that can be used to estimate the age of wild-caught *Ae. albopictus.* We demonstrated the ability of MIRS-ML to age male and female mosquitoes under laboratory as well as under natural conditions, and tested the capacity of the approach to detect age-structure shifts in *Ae. albopictus* populations in a simulated vector control intervention and to provide plausible age structures in field-collected males and females.

## Materials and methods

### Adult *Ae. albopictus* mosquito samples

**Fig. 1:**
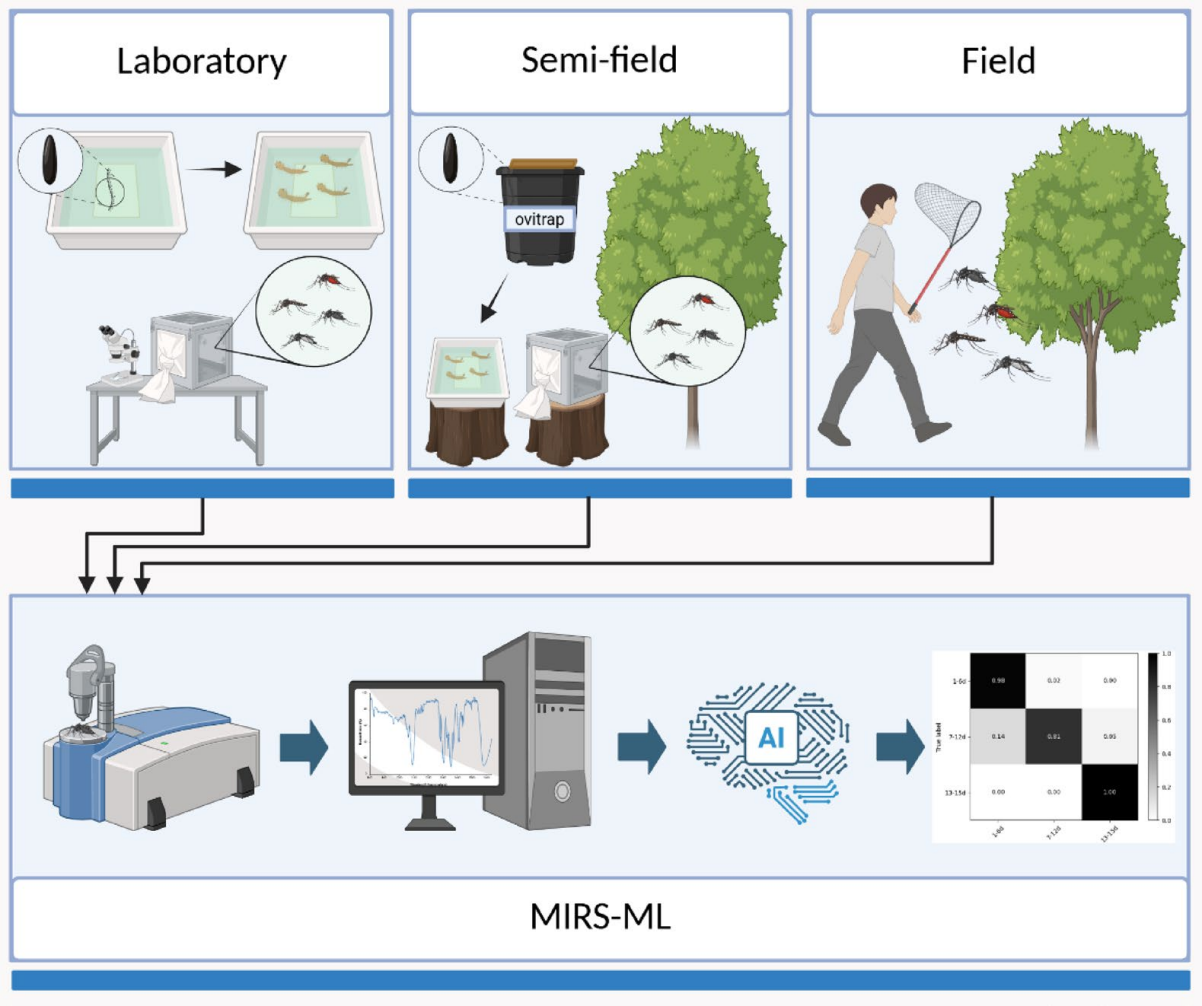
Experimental setup for the development of a MIRS-ML based approach to age-grade *Aedes albopictus* adults. Created in BioRender. della Torre, A. (2025) https://BioRender.com/swrpuj7.

We utilized three sets of *Ae. albopictus* adult samples in this study, i.e. laboratory-reared, semi-field reared and field-collected samples, as follows (Fig. 1).

#### Laboratory-reared samples

Approximately 5,000 dried eggs from an *Ae. albopictus* colony maintained at Sapienza University were allowed to hatch in basins with 2 L of tap water; larvae were reared at a density of 1 larva/mL, and fed with fish food and yeast powder until pupation. Adults emerging in a single day were placed in a 30 cm^3^ cage (300–600 adults/cage) and fed a 10% glucose solution for a total of 4 cages. Mosquitoes (from eggs to adults) were kept at 26 ± 1.0 °C, 60 ± 5% RH, and a 14:10 photoperiod. Males and females were collected on day 1 post-emergence and subsequently every 7 days until day 15 and 36, for males and females, respectively (Table S1). To obtain variability in female gonotrophic stages, females in each cage were provided a blood-meal on different days before the collections. Defibrinated ram blood meals were provided by Hemotek Membrane Feeding System with 3-day intervals, and oviposition cups supplied 2 days after each blood meal. For each age, ~ 25–30 females of each gonotrophic stage—i.e. unfed, freshly fed (1 day Post Bloodmeal, PBM), gravid (2 days PBM), and post egg laying (3 days PBM)—were collected.

Mosquitoes were killed using a chloroform-soaked cotton pad for at least 20 min to preserve the mosquito cuticle for infrared measurements^35^. To avoid measurement alterations due to hydration differences, the mosquitoes were completely dried^21^. For this purpose, specimens were placed in 15 ml Falcon tubes with silica gel behind a cotton barrier and stored at ~ 4 °C until processing with MIRS.

#### Semi-field reared samples

Mosquitoes were obtained from eggs collected via Ovitraps in Aprilia (LT, 41°35′40″ N, 12°39′15″ E) and the Rome metropolitan area (41°53′57.12″ N, 12°32′42.00″ E) in June and July 2023 to ensure a genetically heterogeneous population, following the rational implemented by Siria et al. (2022)^23^. Rearing from hatching to adulthood and adult maintenance occurred at the Experimental Botanical Garden of Sapienza University (Rome, Italy). Approximately 10,000 field-collected eggs were divided into 32 basins (35 × 25 cm), each containing 2 L of water, maintaining a density of ~ 1.5 larvae per ml. The larvae were fed daily with fish food and yeast powder until pupation. Larvae pupated over two consecutive days were separated into plastic cups (approximately 500 pupae/cup) and placed inside cages for emergence, so that each cage had mosquitoes emerging over a three-day period, for a total of 7 cages. Pupae not emerging in this time frame were removed. To obtain variability in female gonotrophic stages, females in each cage were provided a ram blood-meal at different days before collection via a Hemotek Membrane Feeding System brought directly into the semi-field site. Oviposition cups were supplied 2 day after each blood meal. Adults were collected every 3 days to obtain consecutive age classes (1–3, 4–6, 7–9, …, 31–33 days old) (Table S2). Average minimum, maximum and mean temperatures during the experiment were 28.5 °C, 22.6 °C, and 34.3 °C, respectively. Mean relative humidity was 66.4%^36^ and ~ 14:10 photoperiod. Females were collected at various stages: unfed, freshly fed (1 day PBM), gravid (2 days PBM), and post egg laying (3 days PBM). The collection, killing, and storage procedures were consistent with those used for laboratory-reared mosquitoes.

#### Field-collected samples

Between May and June 2023, adult *Ae. albopictus* mosquitoes were sampled using BG traps placed in private gardens in Aprilia (LT, 41°35′40″ N, 12°39′15″ E). Killing and storage procedures were consistent with those used for laboratory- and semi-field-reared specimens.

### Spectroscopy

Mosquito samples were measured using Bruker Alpha II FT-IR spectrophotometers equipped with a diamond ATR crystal at the Vector Biology Group laboratory (University of Glasgow, UK). Only the head and thorax were scanned by placing them at the centre of the crystal and pressing with the anvil attached to the instrument to maximize contact between the mosquito cuticle and the ATR crystal. Background and mid-infrared (MIR) spectra were acquired by averaging 24 scans at a resolution of 4 cm^−1^ over a wavelength range of 4000–400 cm^−1^.

### Machine learning analysis

Low-quality spectra were discarded using a custom script in Python designed for mosquito spectra which consisted of three filters to: 1—eliminate spectra with atmospheric intrusion (CO_2_ and water vapour) by assessing the smoothness of the region between 3900 and 3500 cm^−1^; 2—eliminate low intensity spectra measuring the average absorbance of the plateau in the spectrum between 500 and 400 cm^−1^; 3—eliminate distorted spectra caused by the anvil^21^.

Three datasets of infrared spectra were obtained: one from laboratory-reared individuals, one from semi-field samples, and one from wild populations. The analyses were conducted using Python 3.10 to develop a Supervised Machine Learning (ML) algorithm. The laboratory dataset was used to initially validate the ability of MIRS to detect spectral variations associated with different mosquito ages. The semi-field samples were then used to develop a ML algorithm capable of accurately estimating the age of *Ae. albopictus* individuals. This model was deployed at three different resolution levels, i.e. grouping spectra from mosquitoes emerged in 3, 6 or 9 consecutive days.

For lab and semifield datasets, we shuffled and split the dataset into the training (80%) and test sets (20%), stratified by age groups. The training set was standardised and used to compute baseline performance of eight ML algorithms—LR: Logistic Regression; RF: Random Forest; SVC: Support Vector Classifier; KNN: K-Nearest Neighbours; DT: Decision Tree; GB: Gradient Boosting Classifier; AB: Ada Boost Classifier; ET: Extra Tree Classifier. In all cases, tenfold cross validation and the default parameter settings of the models were used. The best model was chosen based on accuracy as well as its suitability for analysis of high dimensional data. It was then optimised using hyperparameter tuning, which consists of choosing a set of optimal values for the model hyperparameters to maximise its performance. Hyperparameter optimisation was conducted using gridsearch with tenfold cross-validation to identify the best-performing parameter combinations. The selected parameters for our optimised models are reported in Table S3 and Table S4. The remaining 20% of the data (the test set) were used for the final evaluation of the optimised models. Model performance was measured by comparing the estimated age with the known age of the specimens, resulting in a confusion matrix that reported accuracy for each age class. The final optimised model trained in semifield data was used to predict different age classes using data from wild mosquitoes. The analysis was performed using scikit-learn 1.2.2.

### Population simulation and power analysis

To estimate the statistical power of the MIRS-ML model to detect a shift in the age structure of a mosquito population after an insecticidal intervention, we generated computer-simulated mosquito populations under two different scenarios: (i) no intervention and (ii) an adult spray intervention with immediate action of 50% killing efficacy; populations were supposed to be sampled one week after the intervention. The age structure (i.e. the frequency of each age class) in the populations was simulated assuming a constant daily mortality of 4% up to 33 days for females and up to 15 days for males, with no survival after 33- or 15-days post-emergence, respectively. The age structure of each post-intervention population was compared with the control population using Wilcoxon/Mann–Whitney U tests for both females and males (high-resolution algorithm). For males analysed with mid- and low-resolution algorithms, a Fisher exact test was used instead, due to the smaller number of available age classes. Power was estimated as the proportion of 10,000 simulated datasets where a significant (*p* < 0.05) difference in age structure was detected between intervention and control populations across seven sample sizes (n = 20, 50, 100, 150, 200, 250 and 300). Simulations were performed in R v4.3.2.

## Results and discussion

### Mid-infrared spectra of laboratory-reared *Ae. albopictus* varies across ages

**Fig. 2:**
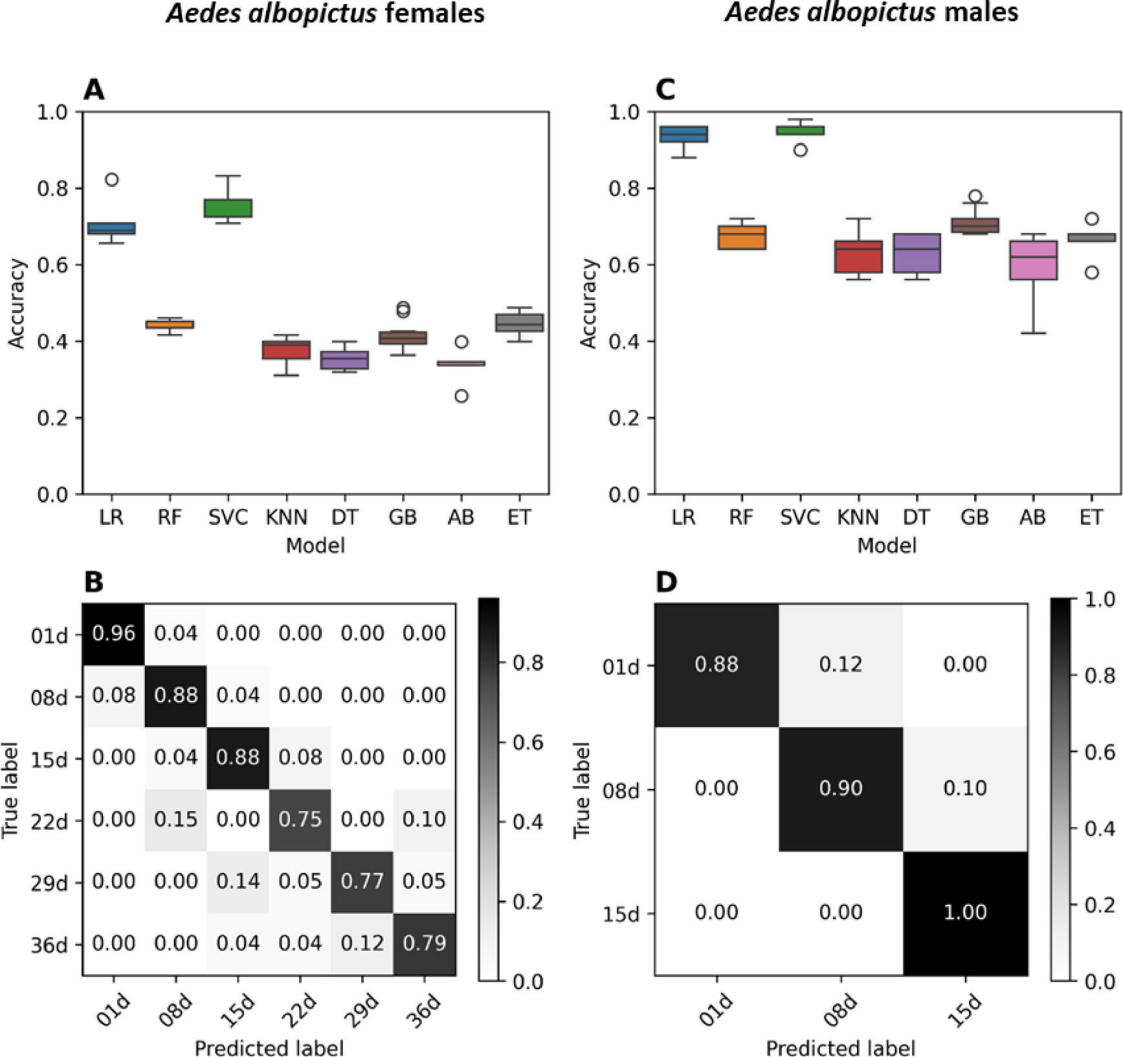
MIRS-ML age prediction for *Ae. albopictus* adults reared in laboratory. (**A**, **C**): Boxplots showing accuracy for each tested model for females (**A**) and males (**C**). LR: Logistic Regression; RF: Random Forest; SVC: Support Vector Classifier; KNN: K-Neighbours; DT: Decision Tree; GB: Gradient Boosting Classifier; AB: Ada Boost Classifier; ET: Extra Tree Classifier. (**B**, **D**): Confusion matrices of age classification for adult females (**B**) and males (**D**). Y axis shows the known ages of mosquitoes, while x axis shows the ages predicted by SVC model. Each cell of the matrix shows the percentage of individuals assigned to each age class. Percentages on the diagonal correspond to individuals whose age was correctly assessed. The colour bar on the left shows the scale of colours that correspond to each accuracy value: from white (0, lowest accuracy) to black (1, highest accuracy).

To test whether MIRS could be used to age-grade *Ae. albopictus* populations, we first applied it to a laboratory dataset. Specifically, we reared and collected 703 female and 316 male adults at different ages (1-, 8-,15-day old for both females and males, and additionally 22-, 29- and 36-day old for females only) and measured them with MIRS (Table S1). We then compared eight different algorithms in terms of their ability to estimate the age of these samples based on their MIR spectra. The models ranked similarly for males and females independently. In both cases, Support Vector Classifier (SVC) had the highest baseline accuracy followed closely by Logistic Regression (LR), which has been used before in similar applications^21^. The baseline accuracies of SVC were 76.1% and 94.8% for females and males, respectively (Fig. 2A, C). As initially the algorithms trained using the whole mid-infrared spectrum (4,000–400 cm^−1^) tended to learn from chemically irrelevant regions (2,500–1,800 cm^−1^, Figure S1), we preselected the region between 1800 and 402 cm^−1^ for the final model. This pre-processing step, also present in similar applications like in Pazmiño-Betancourth et al.^37^, is designed to reduce the noise-to-signal ratio of the spectral data. By restricting the data available to the models to only those regions of the spectra with meaningful chemical information, we reduce the likelihood of overfitting and increase the generalisability of our models.

Prediction accuracy of each age class ranged from 75 to 96% for females (up to 36 days old) and from 88 to 100% for males (up to 15 days old, Fig. 2B, D). The confusion matrix in Fig. 2B also illustrates that, when incorrect, the model generally predicted ages close to the true value. Together, these results demonstrate that the MIR spectra carry a signal that correlates with the age of the individual, and that this signal could be detected by the model, supporting the potential of the MIRS-ML approach as an age-grading method for wild *Ae. albopictus*.

### Models based on MIR spectra from genetically and environmentally variable *Ae. albopictus* can accurately predict age classes

To develop a MIRS-ML approach capable of predicting the age of mosquito field populations, it is essential to train models on spectra from mosquitoes reared outdoors under natural conditions and representing real genetic backgrounds, ensuring that both environmental and genetic variability influencing ageing are properly captured. To this aim, approximately 10,000 field-collected *Ae. albopictus* eggs were reared under semi-field natural conditions; adults were collected at different ages and gonotrophic stages. The final dataset comprised a total of 1,225 and 565 MIR spectra for females and males, respectively (Table S2).

For each sex we developed three machine learning models at high (i.e. successive age classes, each of which including adults emerged over a 3-day period), medium (age classes including adults emerged within 6 days), and low resolution (age classes including adults emerged within 9 days) (Table S5). Similarly to spectra from laboratory reared mosquitoes, SVC had the highest accuracy (Figure S2) and was used for further optimisation.

**Fig. 3:**
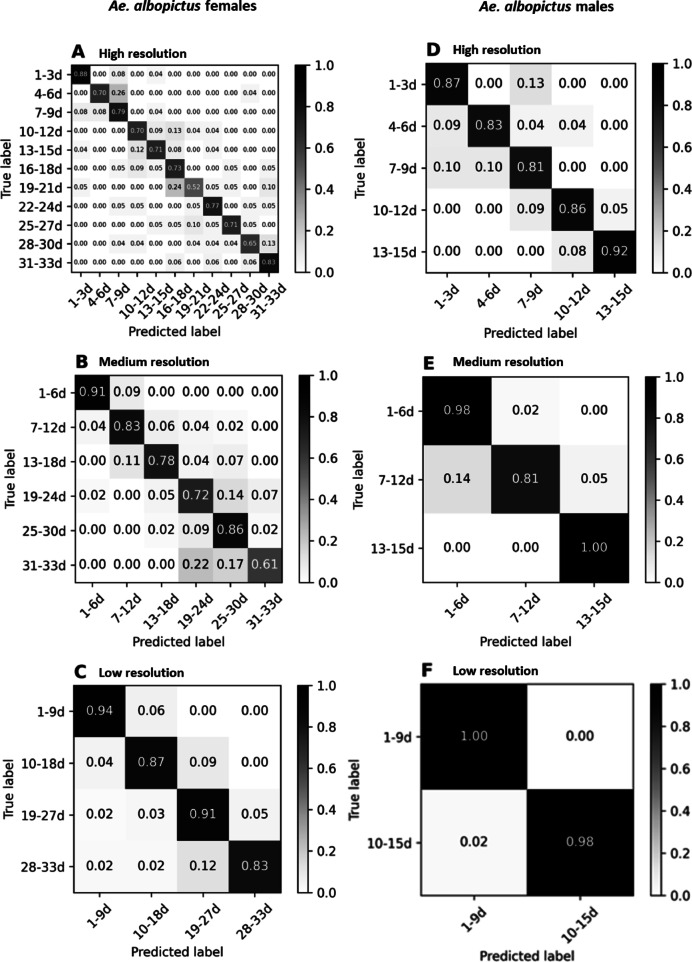
Confusion matrices for three different age classification resolutions for *Ae. albopictus* females (left) and males (right), reared under semi-field conditions. (**A**,**D**): high resolution (3-day classes); (**B**,**E**): medium resolution (6-day classes); (**C,F**): low resolution (9-day classes). Y axes show the true ages of mosquitoes, while x axes show the ages predicted by SVC algorithm. Each cell of the matrix shows the accuracy (between 0 and 1) of individuals assigned to each age class. Percentages on the diagonal correspond to individuals whose age was correctly estimated by the algorithm. The colour bar on the left shows the scale of colours that correspond to each accuracy value: from white (0, lowest accuracy) to black (1, highest accuracy).

The prediction accuracy decreased as the resolution increased. The highest overall model accuracy was obtained with the low-resolution trained model (89.4% for females and 99% for males), then medium-resolution (78.5% for females and 93% for males), and finally the high-resolution (72.6% and 85.8%, respectively) (Fig. 3). Generally, the youngest age class had higher prediction accuracies, suggesting that changes in cuticle composition are larger during the first days after emergence. This was also observed on *Anopheles* malaria vectors^23^. The lower accuracy in predicting female age compared to males may be due to greater physiological variability, as the dataset included unfed, blood-fed and gravid females. Notably, in all cases, but particularly in the high-resolution model, when age classes were misclassified, the model tended to predict the nearest age classes, and not just random ages.

### MIRS machine learning models learned from sex-specific chemical features

**Fig. 4:**
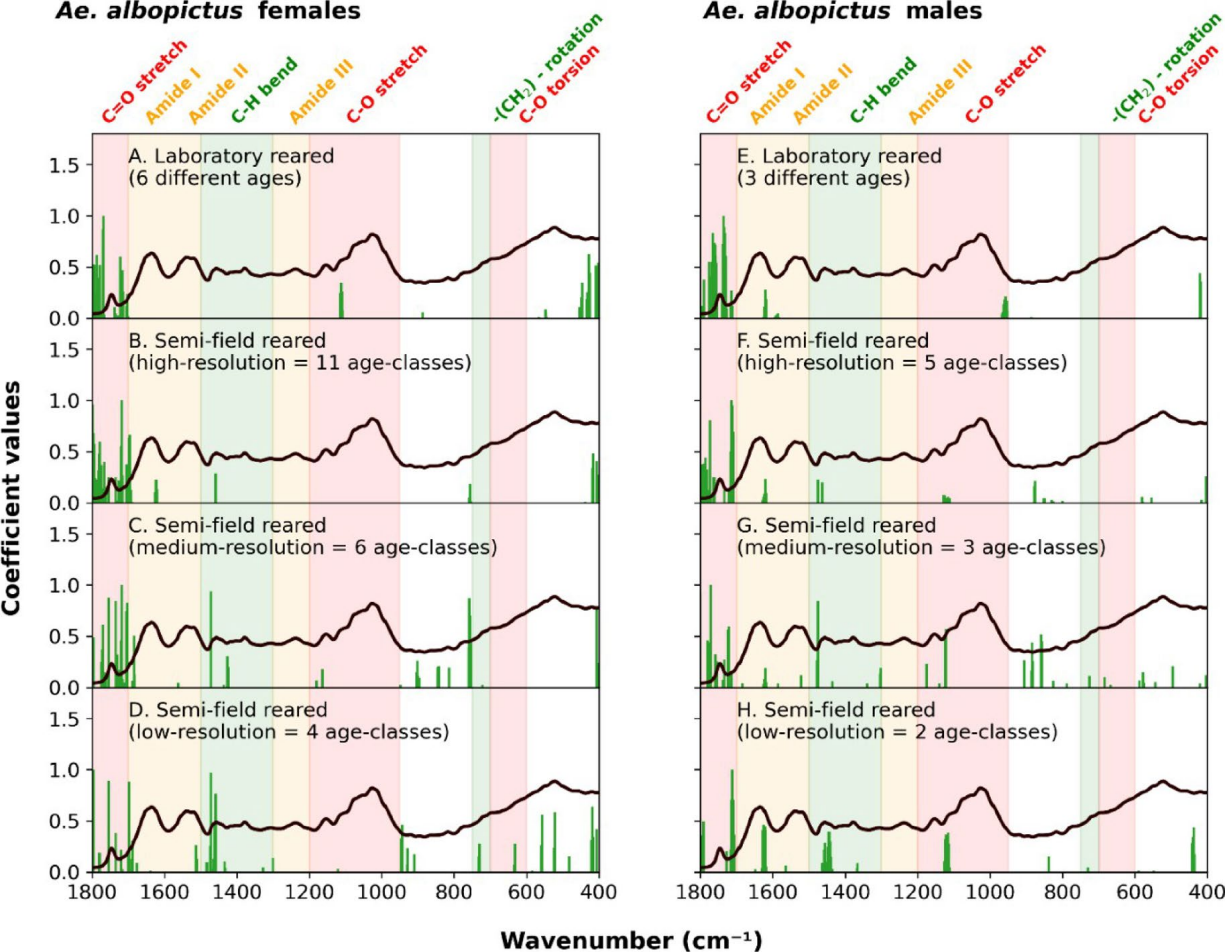
Mean mid-infrared spectrum in the 400–1800 cm^−1^ range of *Aedes albopictus* adult mosquitoes, overlaid with the top 50 most important wavenumbers (features) identified by the ML model. Vertical green lines indicate the wavenumbers with the highest absolute model coefficients values. These features represent the most influential bonds in predicting mosquito age. (**A**) females reared in laboratory; (**B**) semi-field females at high resolution; (**C**) semi-field females at medium resolution; (**D**) semi-field females at low resolution; (**E**) males reared in laboratory; (**F**) semi-field males at high resolution; (**G**) semi-field males at medium resolution; (**H**) semi-field females at low resolution.

To better understand which spectral regions contributed most to the classification, we generated feature importance plots based on the model coefficients (Fig. 4, Table S6, Table S7). The plots display the mean mid-infrared spectrum alongside vertical lines indicating the top 50 wavenumbers with the highest mean absolute coefficient values. These highlighted features represent the spectral regions from which the model learned the most to discriminate between age classes. By comparing the feature importance plots within the same sex, some regions seemed to be consistently influential (Fig. 4).

Features located at 1746 cm^−1^ (corresponding to C=O stretches related to wax, protein and chitin) were the most influential for age classification regardless of sex and rearing conditions (Fig. 4A–H). Similar features have been observed in MIRS-ML models that predict the age of other vectors, specifically in the female malaria mosquitoes *An. gambiae* s.l.^23^ and *An. funestus*^38^ and in the female tsetse flies^39^.

In addition, other wavelengths became influential as the age resolution of semi-field reared samples decreases. In females, these are mostly C-H bend (1457 cm^−1^ and ~ 758 cm^−1^) related to chitin, wax and proteins (Fig. 3B–D). In males (Fig. 3F–H), other biological relevant areas are amide I (1636 cm^−1^, related to chitin and protein) C-H bend (1457 cm^−1^ related to wax and proteins) and C–O stretch (1100 cm^−1^, related to chitin, waxes and proteins).

### MIRS-ML can detect *Ae. albopictus* age structure shift following a simulated intervention

**Fig. 5:**
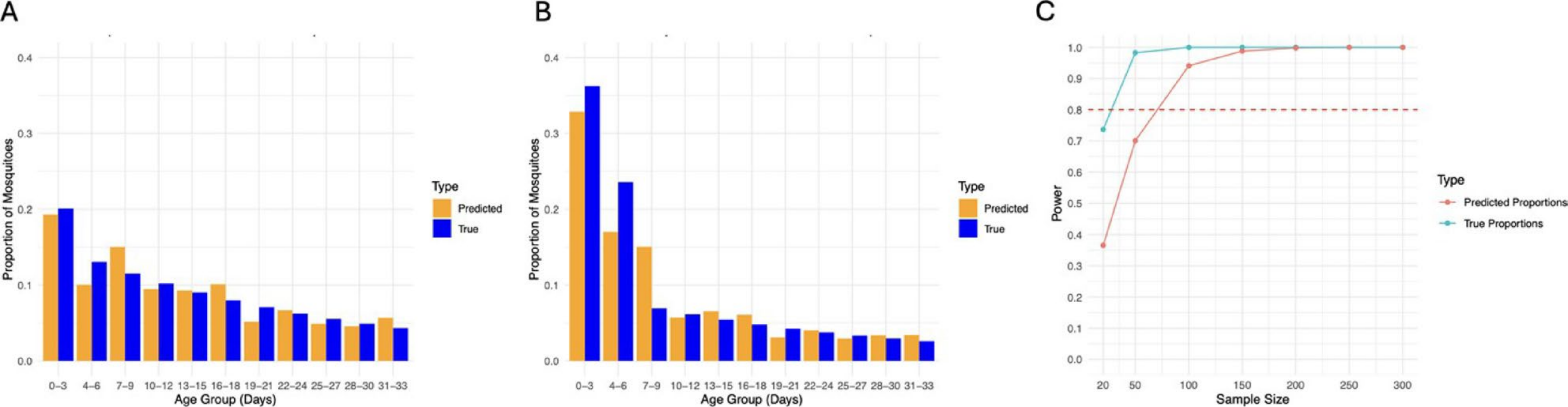
Modelling the assessment of the effectiveness of vector control intervention by shifts in population age structures detected by MIRS-ML. Computer simulations were used to assess the power of MIRS-ML model to detect an age structure shift between (**A**) an *Ae. albopictus* natural population with 4% daily mortality relative to (**B**) a population target of a control intervention killing 50% of the females 1 week earlier. Blue and orange bars indicate the simulated age structure and predicted age structure based on the MIRS-female-high-resolution model, respectively. (**C**) Power to detect an effect of the vector control intervention was estimated over seven sample sizes per population from 20 to 300. The blue line shows the power that would be achieved with 100% accurate age group classification and the red line indicated the power using the MIRS model. The dotted line indicated 80% power at* p* < 0.05.

Age-grading vector populations allows the detection of shifts in mosquito age structures after an intervention, providing a direct way to quantify its impact. We tested if our semi-field MIRS-ML prediction were sufficiently accurate to detect an age-structure shift and compared the models at different age resolutions. First, we simulated the age structure of (1) a control population (4% daily mortality) and (2) a population sampled one week after an adulticide intervention of 50% efficacy. Then, we applied our MIRS-ML models to reconstruct the predicted proportions of different age classes and compared with 100% correct predictions (Fig. 5A,B, Figure S3A-E). Finally, we estimated the statistical power of MIRS-ML to detect a shift in the different age classes expected from the intervention (Fig. 5C, Figure S3C-F). When applying MIRS-ML on females using the high-resolution age classes, sampling less than 100 mosquitoes pre- and post-intervention was sufficient to obtain > 80% power to detect an age structure shift; however, when using medium- and low-resolution classes a larger sample size was required to achieve > 80% power, showing that predicting more age classes, even if with overall lower accuracy, is the most powerful approach to detect a population age structure shift following a vector control intervention. Similar results were obtained when the models were applied to male mosquito populations (Figure S4).

### MIRS-ML predicted plausible age structures from both laboratory-reared and wild collected *Ae. albopictus* female and male adults

**Fig. 6:**
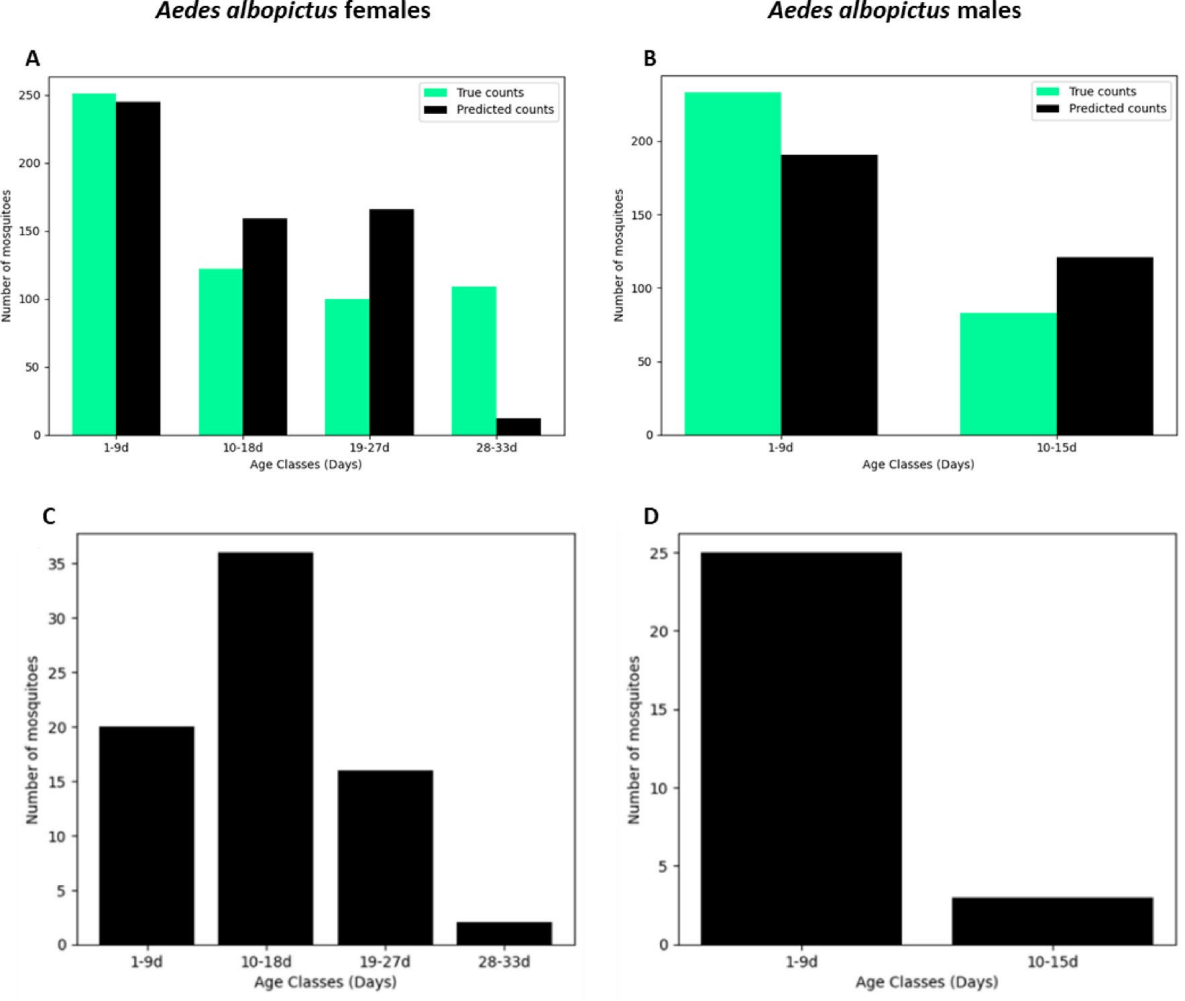
MIRS-ML age structures predictions of lab and wild collected *Aedes albopictus* adults. Barplots show the number of *Ae. albopictus* lab females (**A**) and males (**B**) and wild females (**C**) and males (**D**) assigned to different age classes based on MIRS-ML low resolution model. (**A**) and (**B**): laboratory-reared females were 1, 8, 15, 22 and 18 days old; laboratory-reared males were 1, 8 and 15 days old. (**C**) and (**D**): females and males of unknown ages.

We then evaluated the generalisability of the low-resolution semi-field MIRS-ML model by testing it on two independent, previously unseen datasets. This test was not carried out with the higher resolution models because the validation dataset lacked consistent age group representation, making a full validation impossible. First, the model was applied to the laboratory dataset. Notably, the model correctly assigned most of 1 and 8 day old females to the first age class (1–9 d), overestimated the assignment of 15 and 22 old females to the intermediate age classes, and largely underestimates the assignment of 29d old females to the oldest age class (Fig. 6A). In the case of males—for which only 2 age classes were considered—the model underestimated the younger class (Fig. 6B). One possible explanation for the underestimation of age in laboratory-reared females is that the semi-field MIRS-ML model was trained on females exposed to more natural environmental conditions including warmer temperatures and larger daily thermal excursion, which likely accelerated their biological ageing. As a result, older laboratory-reared females may be biologically younger than their semi-field counterparts of the same chronological age. A similar pattern was reported in *Anopheles gambiae s.l.* females, where models trained on laboratory data overestimated the age of semi- field mosquitoes^23^. The reason for the overestimation of age in laboratory-reared males is less clear. One possibility is that males aged more rapidly in the laboratory due to more frequent mating opportunities, which are typical in colony conditions, although specific studies are needed to support this hypothesis.

Then, we tested the model on a limited sample of 74 females and 28 *Ae. albopictus* males collected in the field near Rome, which, having experienced natural climatic conditions, could be expected to exhibit more comparable ageing rates.

As determining the number of gonotrophic cycles by examining the ovariole dilatations is not feasible in this species, our test simply aimed at assessing if the predicted age structure would be realistic in field populations. Our predictions reconstructed age structures that are plausible in relation to the expected ones (Fig. 6C, D). In females, the majority (48%) was classified as medium old (10–18 day old), the rest as either younger or older (49% combining 1–9 days and 19–27 days) and a very small proportion (3%) as the oldest age class (28–33 days). In males, the large majority (89%) was classified as the youngest age class (1–9 day old). These results also have implications for disease transmission, as age estimation of female mosquitoes can help predict the risk of arboviral spread, since the extrinsic incubation period of dengue virus (DENV) can last several days^40^.

## Conclusion

The results obtained represent a first relevant step towards the development of a sound and reproducible MIRS-ML approach for age-grading of *Ae. albopictus* populations in their invasive range. First, we demonstrated the ability of MIRS-ML to age-grade male and female mosquitoes under laboratory conditions. Second, we optimised the model with mosquitoes reared under natural conditions in a semi-field facility, in order to expose them to more realistic ambient conditions. Then, we showed that the models are capable of detecting age-structure shifts in *Ae. albopictus* populations in a simulated vector control intervention. Finally, we validated MIRS-ML on unseen data and reconstructed plausible age structures in field-collected *Ae. albopictus* male and female mosquitoes.

Future work will focus on improving the method’s accuracy and generalisability by expanding the training datasets with spectra from more diverse mosquito populations and incorporating environmental variables known to influence mosquito ageing. While our developed model demonstrated some generalisability when applied to unseen datasets—including both laboratory-reared and field-caught *Ae. albopictus* mosquitoes - it has not yet been tested on more genetically and environmentally diverse samples. Since MIRS detects age- related changes in the mosquito cuticle, variation in genetic background or environmental conditions may affect the resulting spectra and, consequently, the performance of MIRS-ML models. In our study, the field validation—though it produced plausible age structures—was based on a population genetically similar to the one used for training. Moreover, both the training and field-sampled mosquitoes were collected from the same region and during the same season, likely experiencing similar environmental conditions. Therefore, while our results are promising, the current MIRS-ML models require broader validation and refinement across diverse populations and ecological contexts to become a robust tool for global vector surveillance. Due to the low processing costs, we expect this approach to have the potential to be largely exploited in the future to characterise the age structure of *Ae. albopictus* field populations for improved epidemiological risk models and for assessment of the effectiveness of mosquito control interventions. This is particularly valuable in non-endemic settings where *Ae. albopictus* has become established, as the impact of interventions cannot be directly measured through changes in disease prevalence. By focusing on the age structure of the population in an area treated with vector-control, it would be possible to gain a deeper understanding of how adulticide interventions affect mosquito populations over time and their contribution to target the older, disease-transmitting vectors, providing a more comprehensive assessment of their effectiveness.

## Supplementary Information

Below is the link to the electronic supplementary material.

Supplementary Material 1

## Data Availability

Data can be accessed at: https://github.com/casasgomezuribarri/Foti_et_al_albopictus_DATA. Scripts can be accessed at: https://github.com/maurocolapso/AlbopictusMIRS_Foti_et_al_2025.git

## References

[1] Wagner, I. et al. Rapid identification of mosquito species and age by mass spectrometric analysis. *BMC Biol.* **21**, 10 (2023). 36690979 10.1186/s12915-022-01508-8 PMC9872345

[2] Lopez, K., Irwin, P., Bron, G. M., Paskewitz, S. & Bartholomay, L. Ultra-low volume (ULV) adulticide treatment impacts age structure of *Culex* species (Diptera: Culicidae) in a West Nile virus hotspot. *J. Med. Entomol.* **60**, 1108–1116 (2023). 37473814 10.1093/jme/tjad088

[3] Pautzke, K. C. *Using age structure to assess adulticide efficacy on Culex mosquitoes in Maricopa county* (University of Arizona, Tucson, 2020).

[4] Beklemishev, W. N., Detinova, T. S. & Polovodova, V. P. Determination of physiological age in anophelines and of age distribution in anopheline populations in the USSR. *Bull. World Health Organ.* **21**, 223–232 (1959). 13798390 PMC2537872

[5] Detinova, T. S., Bertram, D. S. & Organization, W. H. *Age-Grouping Methods in Diptera of Medical Importance, with Special Reference to Some Vectors of Malaria*. (World Health Organization, 1962). 13885800

[6] Hugo, L., Monkman, J. & Dave, K. Proteomic biomarkers for ageing the Mosquito *Aedes aegypti* to determine risk of pathogen transmission. *ResearchGate* (2013). 10.1371/journal.pone.0058656 PMC3594161 23536806

[7] Hugo, L. E., Quick-miles, S., Kay, B. H. & Ryan, P. A. Evaluations of Mosquito age grading techniques based on morphological changes. *J. Med. Entomol.* **45**, 353–369 (2008). 18533427 10.1603/0022-2585(2008)45[353:eomagt]2.0.co;2

[8] Johnson, B. J., Hugo, L. E., Churcher, T. S., Ong, O. T. W. & Devine, G. J. Mosquito age grading and vector-control programmes. *Trends Parasitol.* **36**, 39–51 (2020). 31836285 10.1016/j.pt.2019.10.011

[9] Wang, M.-H. et al. Gene expression-based biomarkers for *Anopheles gambiae* age grading. *PLoS ONE* **8**, e69439 (2013). 23936017 10.1371/journal.pone.0069439 PMC3720620

[10] Sikulu, M. T. et al. Proteomic changes occurring in the malaria mosquitoes *Anopheles gambiae* and *Anopheles stephensi* during aging. *J. Proteomics* **126**, 234–244 (2015). 26100052 10.1016/j.jprot.2015.06.008

[11] Cook, P. E. et al. The use of transcriptional profiles to predict adult mosquito age under field conditions. *Proc. Natl. Acad. Sci.* **103**, 18060–18065 (2006). 17110448 10.1073/pnas.0604875103 PMC1838706

[12] Iovinella, I., Caputo, B. & Michelucci, E. Candidate biomarkers for mosquito age-grading identified by label-free quantitative analysis of protein expression in *Aedes albopictus* females. *J. Proteomics* **128**, 272–279 (2015). 26271156 10.1016/j.jprot.2015.08.002

[13] Weeratunga, P., Rodrigo, C., Fernando, S. D. & Rajapakse, S. Control methods for *Aedes albopictus* and *Aedes aegypti*. *Cochrane Database Syst. Rev.* **2017**, CD12759 (2017).

[14] Cook, P. E. et al. Predicting the age of mosquitoes using transcriptional profiles. *Nat. Protoc.* **2**, 2796–2806 (2007). 18007615 10.1038/nprot.2007.396

[15] Costa, M. M., Corbel, V., Ben Hamouda, R. & Almeras, L. MALDI-TOF MS profiling and its contribution to mosquito-borne diseases: A systematic review. *Insects* **15**, 651 (2024). 39336619 10.3390/insects15090651 PMC11432722

[16] Mayagaya, V. S. et al. Non-destructive determination of age and species of *Anopheles gambiae s.l.* using near-infrared spectroscopy. *Am. J. Trop. Med. Hyg.* **81**, 622–630 (2009). 19815877 10.4269/ajtmh.2009.09-0192

[17] Stuart, B. H. *Infrared Spectroscopy: Fundamentals and Applications*. (2004).

[18] Baker, M. J. et al. Using Fourier transform IR spectroscopy to analyze biological materials. *Nat. Protoc.* **9**, 1771–1791 (2014). 24992094 10.1038/nprot.2014.110 PMC4480339

[19] Mwanga, E. P. et al. Using mid-infrared spectroscopy and supervised machine-learning to identify vertebrate blood meals in the malaria vector, *Anopheles arabiensis*. *Malar. J.* **18**, 187 (2019). 31146762 10.1186/s12936-019-2822-y PMC6543689

[20] Fernandes, J. N. et al. Rapid, noninvasive detection of Zika virus in *Aedes aegypti* mosquitoes by near-infrared spectroscopy. *Sci. Adv.* **4**, eaat496 (2018). 10.1126/sciadv.aat0496 PMC5966221 29806030

[21] González Jiménez, M. et al. Prediction of mosquito species and population age structure using mid-infrared spectroscopy and supervised machine learning. *Wellcome Open Res.* **4**, 76 (2019). 31544155 10.12688/wellcomeopenres.15201.1 PMC6753605

[22] Milali, M. P. et al. Age grading *An. gambiae* and *An. arabiensis* using near infrared spectra and artificial neural networks. *PLoS ONE* **14**, e0209451 (2019). 31412028 10.1371/journal.pone.0209451 PMC6693756

[23] Siria, D. J. et al. Rapid age-grading and species identification of natural mosquitoes for malaria surveillance. *Nat. Commun.* **13**, 1501 (2022). 35314683 10.1038/s41467-022-28980-8 PMC8938457

[24] Mwanga, E. P. et al. Detection of malaria parasites in dried human blood spots using mid-infrared spectroscopy and logistic regression analysis. *Mal. J.* **18**, 341 (2019). 10.1186/s12936-019-2982-9 PMC6781347 31590669

[25] Mshani, I. H. et al. Screening of malaria infections in human blood samples with varying parasite densities and anaemic conditions using AI-Powered mid-infrared spectroscopy. *Malar. J.* **23**, 188 (2024). 38880870 10.1186/s12936-024-05011-z PMC11181574

[26] Mwanga, E. P. et al. Rapid assessment of the blood-feeding histories of wild-caught malaria mosquitoes using mid-infrared spectroscopy and machine learning. *Malar. J.* **23**, 86 (2024). 38532415 10.1186/s12936-024-04915-0 PMC10964711

[27] Sikulu, M. et al. Near-infrared spectroscopy as a complementary age grading and species identification tool for African malaria vectors. *Parasit. Vectors* **3**, 49 (2010). 20525305 10.1186/1756-3305-3-49 PMC2902455

[28] Khoshmanesh, A. et al. Screening of *Wolbachia* endosymbiont infection in *Aedes aegypti* mosquitoes using attenuated total reflection mid-infrared spectroscopy. *Anal. Chem.* **89**, 5285–5293 (2017). 28332822 10.1021/acs.analchem.6b04827

[29] Pazmiño-Betancourth, M. *Towards a Portable Mid-Infrared Tool for Analysis of Mosquito Vectors of Malaria* (University of Glasgow, Glasgow, 2023). 10.5525/gla.thesis.83455.

[30] Rezza, G. et al. Infection with chikungunya virus in Italy: An outbreak in a temperate region. *Lancet* **370**, 1840–1846 (2007). 18061059 10.1016/S0140-6736(07)61779-6

[31] La Ruche, G. et al. First two autochthonous dengue virus infections in metropolitan France, September 2010. *Euro Surveill* **15**, 19676 (2010). 20929659

[32] Gjenero-Margan, I. et al. Autochthonous dengue fever in Croatia, August-September 2010. *Euro Surveill* **16**, 19805 (2011). 21392489

[33] Sikulu-Lord, M. T., Devine, G. J., Hugo, L. E. & Dowell, F. E. First report on the application of near-infrared spectroscopy to predict the age of *Aedes albopictus* Skuse. *Sci. Rep.* **8**, 9590 (2018). 29941924 10.1038/s41598-018-27998-7 PMC6018420

[34] Ong, O. T. W. et al. Ability of near-infrared spectroscopy and chemometrics to predict the age of mosquitoes reared under different conditions. *Parasit. Vectors* **13**, 160 (2020). 32228670 10.1186/s13071-020-04031-3 PMC7106667

[35] Mgaya, J. N. et al. Effects of sample preservation methods and duration of storage on the performance of mid-infrared spectroscopy for predicting the age of malaria vectors. *Parasit. Vectors* **15**, 281 (2022). 35933384 10.1186/s13071-022-05396-3 PMC9356448

[36] My OpenLayers Map. https://scia.isprambiente.it/servertsutm/serietemporali400.php.

[37] Pazmiño-Betancourth, M. et al. Evaluation of diffuse reflectance spectroscopy for predicting age, species, and cuticular resistance of *Anopheles gambiae s.l* under laboratory conditions. *Sci. Rep.* **13**, 18499 (2023). 37898634 10.1038/s41598-023-45696-x PMC10613238

[38] Mwanga, E. P. et al. Rapid classification of epidemiologically relevant age categories of the malaria vector, *Anopheles funestus*. *Parasit. Vectors* **17**, 143 (2024). 38500231 10.1186/s13071-024-06209-5 PMC10949582

[39] Pazmiño-Betancourth, M. et al. Advancing age grading techniques for *Glossina morsitans morsitans*, vectors of African trypanosomiasis, through mid-infrared spectroscopy and machine learning. *Biol. Methods Protocols* **9**, bpae058 (2024). 10.1093/biomethods/bpae058 PMC11407438 39290986

[40] Tjaden, N. B., Thomas, S. M., Fischer, D. & Beierkuhnlein, C. Extrinsic incubation period of dengue: Knowledge, backlog, and applications of temperature dependence. *PLoS Negl. Trop. Dis.* **7**, e2207 (2013). 23826399 10.1371/journal.pntd.0002207 PMC3694834

