# Supplementary material for: Towards scalable age-grading of Aedes albopictus mosquito using mid-infrared spectroscopy and machine learning
Source: Sci Rep. 2025 Nov 18;15:40470. doi: 10.1038/s41598-025-24404-x (PMC12627515; doi:10.1038/s41598-025-24404-x)
Supplement: Supplementary file 1 — Supplementary Material 1 [file 41598_2025_24404_MOESM1_ESM.docx]

**Supplementary Information**

**Towards Scalable Age-Grading of *Aedes albopictus* mosquito using Mid-Infrared Spectroscopy and Machine Learning**

Mattia Foti^1^, Martina Micocci^1^, Mauro Pazmiño-Betancourth^2^, Ivan Casas Gomez-Uribarri^2^, Paola Serini^1^, Beniamino Caputo^1^, Alessandra della Torre^1,5*^, Francesco Baldini^2,3,4*^.

1. University of Rome La Sapienza
2. School of Biodiversity, One Health, and Veterinary Medicine, University of Glasgow, Glasgow, UK
3. Department of Environmental Health and Ecological Sciences, Ifakara Health Institute, Ifakara, Tanzania
4. [Francesco.Baldini@glasgow.ac.uk](mailto:Francesco.Baldini@glasgow.ac.uk)
5. [alessandra.dellatorre@uniroma1.it](mailto:alessandra.dellatorre@uniroma1.it)

*These authors equally supervised the work

**Supplementary Figures**

**Fig. S1:** **Whole mean mid-infrared spectrum** **(400-4000 cm^-1^) of *Aedes albopictus* adult** **mosquitoes reared in laboratory**, overlaid with the top 50 most important wavenumbers (features) identified by the ML model.


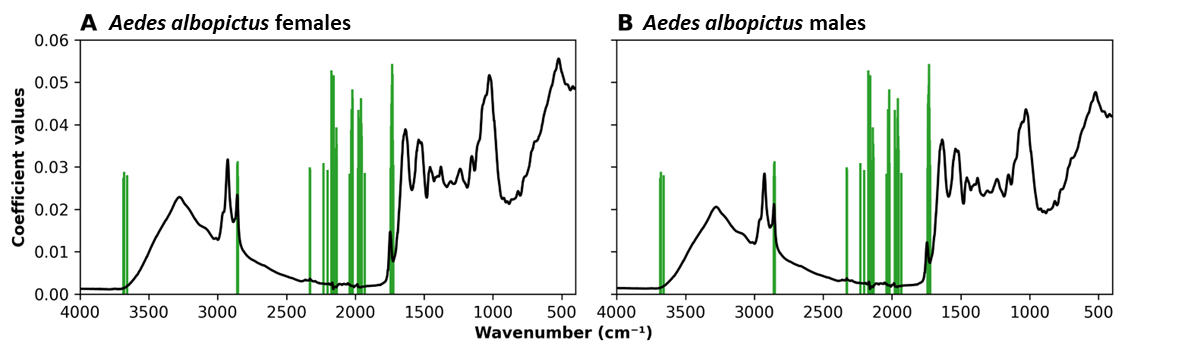


**Fig. S2: Boxplots showing accuracy for each tested model** for females (A) and males (B) reared under semi-field conditions. Each plot refers to a different resolution of age-grading: high resolution (left): 3-days long classes; medium resolution (middle): 6-days long classes; low resolution (right): 9-days long classes. LR: Logistic Regression; RF: Random Forest; SVC: Support Vector Classifier; KNN: K-Neighbours; DT: Decision Tree; GB: Gradient Boosting Classifier; AB: Ada Boost Classifier; ET: Extra Tree Classifier.


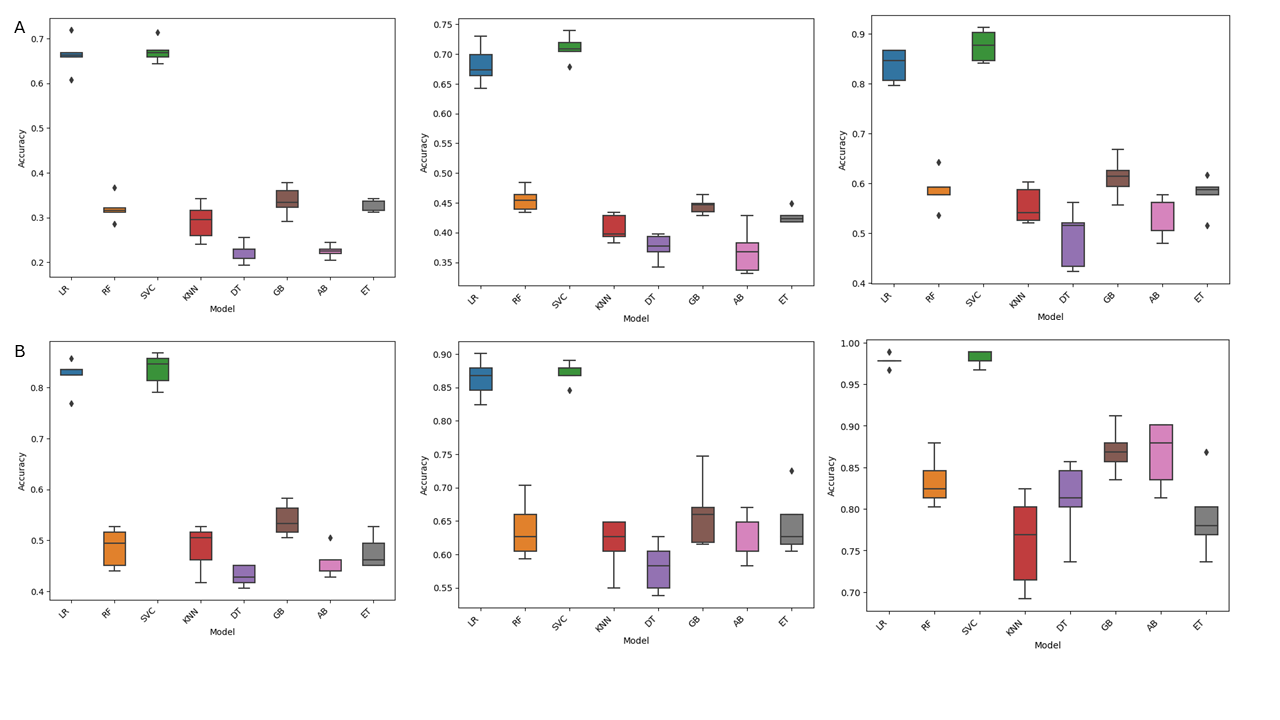


**Figure S3 - ﻿Detection of vector control intervention using MIRS-ML medium and low-resolution models in females**. Computer simulations were used to assess the power of MIRS-ML model to detect an age structure shift between **A, D)** an Ae. albopictus natural population with 0.04% daily mortality relative to **B, E)** a population target of a control intervention killing 50% of the females 1 week earlier. Blue and orange bars indicate the simulated age structure and predicted age structure, respectively, based on the MIRS-female medium-resolution (**A, B**) and low resolution (**D, E**). **C, F)** Power to detect an effect of the vector control intervention was estimated over seven sample sizes per population from 20 to 300. The blue line shows the power that would be achieved with 100% accurate age group classification and the red line indicated the power using the MIRS model. **C** and **F** refer to medium and low resolution, respectively. The dotted line indicated 80% power at p<0.05.

**
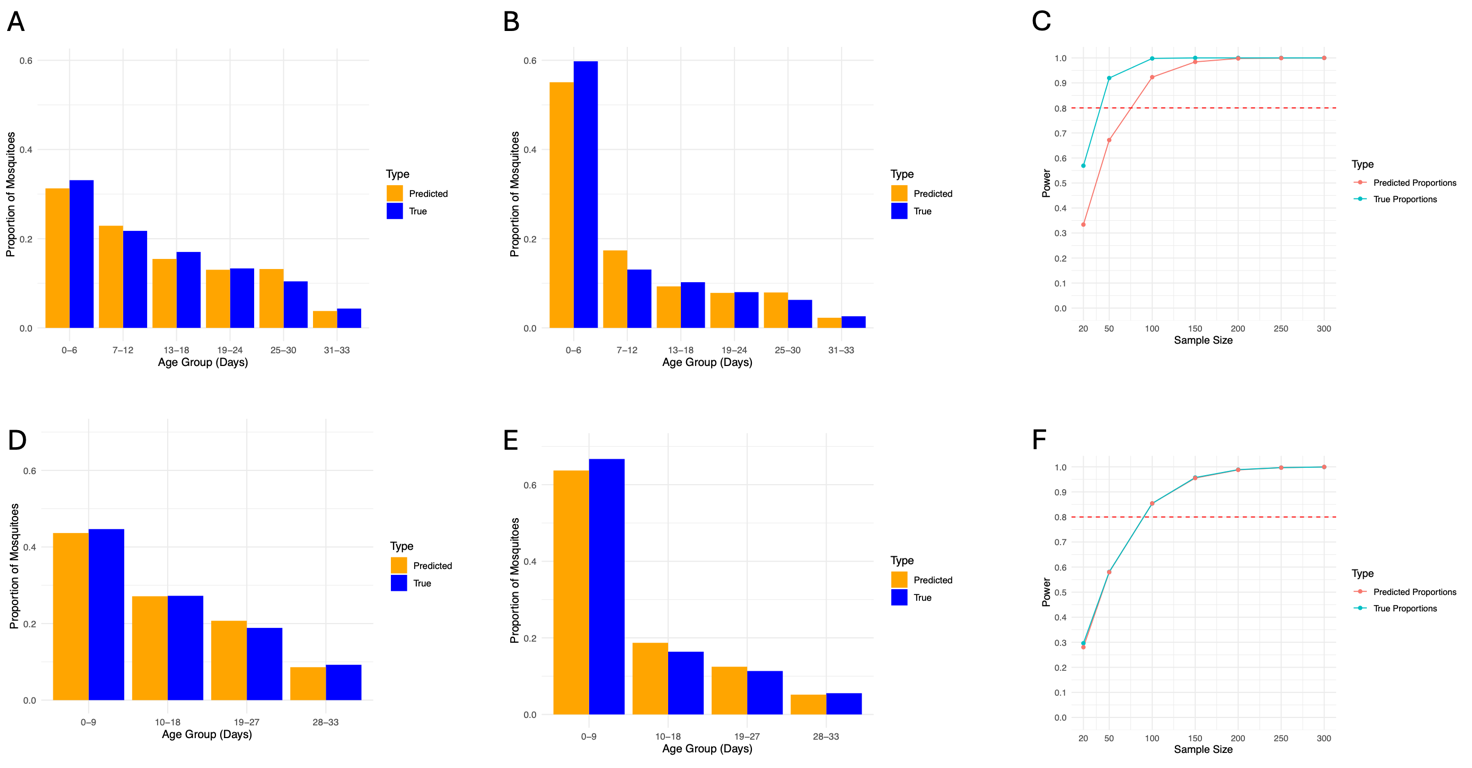
**

**Figure S4 - ﻿Detection of vector control intervention using MIRS-ML models in males**. Computer simulations were used to assess the power of MIRS-ML model to detect an age structure shift between **A, D, G)** an Ae. albopictus natural population with 0.04% daily mortality relative to **B, E, H)** a population target of a control intervention killing 50% of the females 1 week earlier. Blue and orange bars indicate the simulated age structure and predicted age structure, respectively, based on the MIRS-male high resolution (**A, B**) medium-resolution (**D, E**) and low resolution (**G, H**). **C, F, I)** Power to detect an effect of the vector control intervention was estimated over seven sample sizes per population from 20 to 300. The blue line shows the power that would be achieved with 100% accurate age group classification and the red line indicated the power using the MIRS model. **C**, **F** and **I** refer to high, medium and low resolution, respectively. The dotted line indicated 80% power at p<0.05.


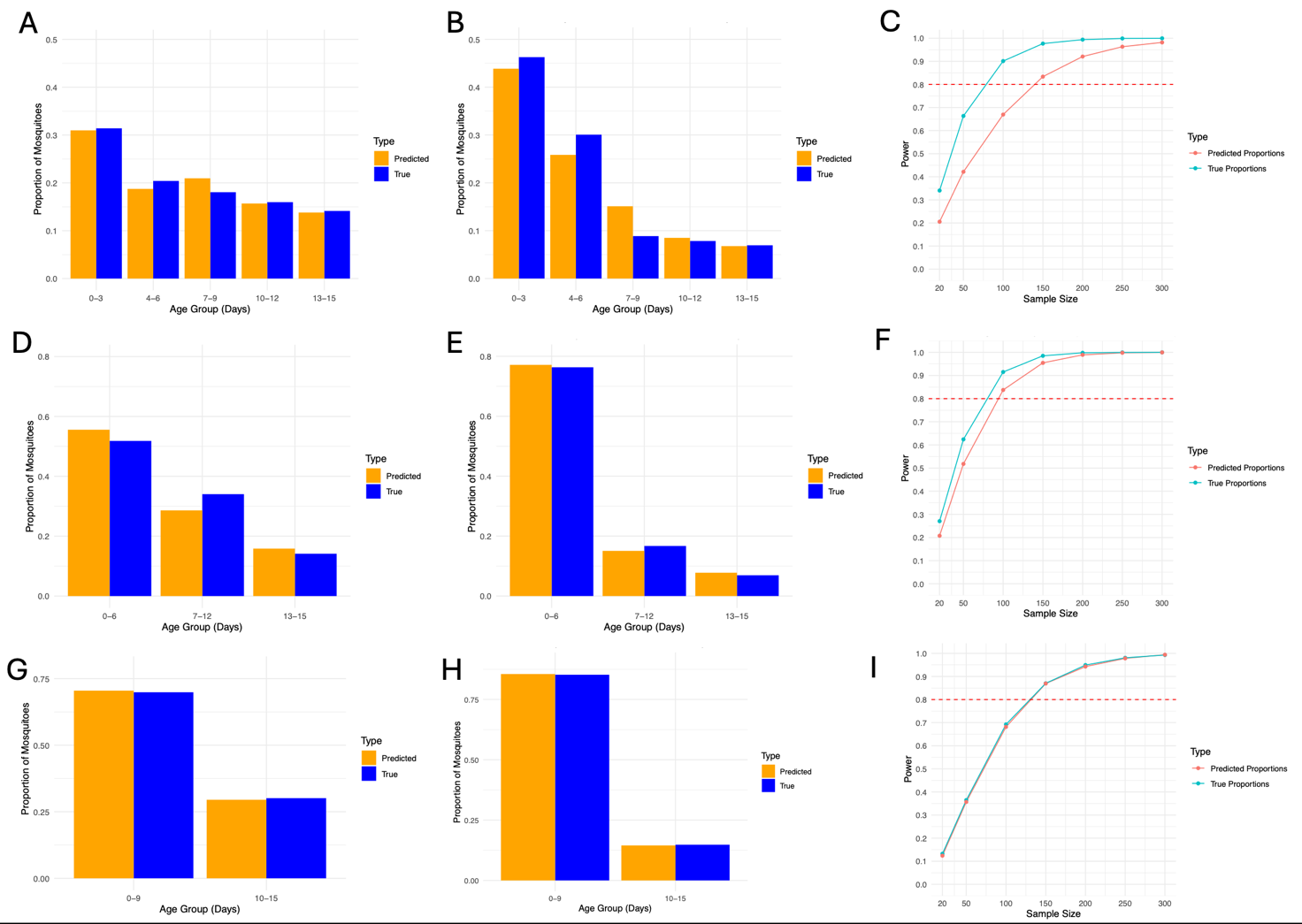


**SUPPLEMENTARY TABLES**

**Table S1**. Number of laboratory reared *Aedes albopictus* adults of different ages used for development of MIRS-ML age-grading approach.

| **Days after emergence** | **N° Females** | **N° Males** |
| --- | --- | --- |
| 1 | 124 | 127 |
| 8 | 127 | 106 |
| 15 | 122 | 83 |
| 22 | 100 | - |
| 29 | 109 | - |
| 36 | 121 | - |
| Total n° of samples | 703 | 316 |

**Table S2**. Number of semi-field reared *Aedes albopictus* adults subdivided grouped in age-classes including individuals emerged in three consecutive days used for development of MIRS-ML age-grading approach.

| **Days after emergence** | **N° Females** | **N° Males** |
| --- | --- | --- |
| 1-2-3 | 120 | 116 |
| 4-5-6 | 116 | 116 |
| 7-8-9 | 118 | 105 |
| 10-11-12 | 115 | 107 |
| 13-14-15 | 118 | 121 |
| 16-17-18 | 111 | - |
| 19-20-21 | 107 | - |
| 22-23-24 | 109 | - |
| 25-26-27 | 106 | - |
| 28-29-30 | 113 | - |
| 31-32-33 | 92 | - |
| Total n° of samples | 1225 | 565 |

**Table S3**. Optimized hyperparameters (C, Gamma, Kernel) for the Support Vector Classifier models used to age-grade Aedes albopictus adults reared in laboratory

|  | **Females** | **Males** |
| --- | --- | --- |
| **C** | 100 | 1.0 |
| **Gamma** | 0.1 | 0.1 |
| **Kernel** | Linear | Linear |

**Table S4.** Optimized hyperparameters (C, Gamma, Kernel) for the Support Vector Classifier models used to age-grade at different resolutions Aedes albopictus adults reared in semi-field.

|  | **High resolution** | | **Medium resolution** | | **Low resolution** | |
| --- | --- | --- | --- | --- | --- | --- |
|  | **Females** | **Males** | **Females** | **Males** | **Females** | **Males** |
| **C** | 100 | 10 | 100 | 100 | 100 | 100 |
| **Gamma** | 0.1 | 0.1 | 0.1 | 0.1 | 0.1 | 0.1 |
| **Kernel** | Linear | Linear | Linear | Linear | Linear | Linear |

**Table S5.** Sample size of *Aedes albopictus* females and males for each resolution of age estimation (High, Medium and Low resolution.

| **High resolution** | | **Medium resolution** | | **Low resolution** | |
| --- | --- | --- | --- | --- | --- |
| **Females age-classes** | **Males age-classes** | **Females age-classes** | **Males age-classes** | **Females age-classes** | **Males age-classes** |
| 1-3 days old | 1-3 days old | 1-6 days old | 1-6 days old | 1-9 days old | 1-9 days old |
| 4-6 days old | 4-6 days old | 7-12 days old | 7-12 days old | 10-18 days old | 10-15 days old |
| 7-9 days old | 7-9 days old | 13-18 days old | 13-15 days old | 19-27 days old | - |
| 10-12 days old | 10-12 days old | 19-24 days old | - | 28-33 days old | - |
| 13-15 days old | 13-15 days old | 25-30 days old | - | - | - |
| 16-18 days old | - | 31-33 days old | - | - | - |
| 19-21 days old | - | - | - | - | - |
| 22-24 days old | - | - | - | - | - |
| 25-27 days old | - | - | - | - | - |
| 28-30 days old | - | - | - | - | - |
| 31-33 days old | - | - | - | - | - |

**Table S6. The 50 wavenumbers from which the *Aedes albopictus* females’ algorithms learn the most, along with their corresponding weight coefficients. The wavenumbers** are listed in ascending order. The weight coefficients indicate the relative contribution of each wavenumber in classifying mosquito age using SVC algorithm

**Table S7. The 50 wavenumbers from which the *Aedes albopictus* males’ algorithms learn the most, along with their corresponding weight coefficients.** The wavenumbers are listed in ascending order. The weight coefficients indicate the relative contribution of each wavenumber in classifying mosquito age using SVC algorithm.
